# Silencing of the small GTPase DIRAS3 induces cellular senescence in human white adipose stromal/progenitor cells

**DOI:** 10.18632/aging.101197

**Published:** 2017-03-17

**Authors:** Asim Ejaz, Monika Mattesich, Werner Zwerschke

**Affiliations:** ^1^ Division of Cell Metabolism and Differentiation Research, Institute for Biomedical Aging Research, University of Innsbruck, A-6020 Innsbruck, Austria; ^2^ Department of Plastic and Reconstructive Surgery, Innsbruck Medical University, A-6020 Innsbruck, Austria

**Keywords:** adipose stem cell, adipose stromal/progenitor cell, adipocyte, adipogenesis, aging, DIRAS3, mTOR, obesity, senescence

## Abstract

Inhibition of Akt-mTOR signaling protects from obesity and extends life span in animals. In the present study, we analyse the impact of the small GTPase, GTP-binding RAS-like 3 (DIRAS3), a recently identified weight-loss target gene, on cellular senescence in adipose stromal/progenitor cells (ASCs) derived from human subcutaneous white adipose tissue (sWAT). We demonstrate that DIRAS3 knock-down (KD) in ASCs induces activation of Akt-mTOR signaling and proliferation arrest. DIRAS3 KD ASCs lose the potential to form colonies and are negative for Ki-67. Moreover, silencing of DIRAS3 results in a premature senescence phenotype. This is characterized by senescence-associated *β*-galactosidase positive enlarged ASCs containing increased p16^INK4A^ level and activated retinoblastoma protein. DIRAS3 KD ASCs form senescence-associated heterochromatic foci as shown by increased level of γ-H2A.X positive foci. Furthermore, these cells express a senescence-associated secretory phenotype characterized by increased interleukin-8 secretion. Human DIRAS3 KD ASCs develop also a senescence phenotype in sWAT of SCID mice. Finally, we show that DIRAS3 KD in ASCs stimulates both adipogenic differentiation and premature senescence. In conclusion, our data suggest that silencing of DIRAS3 in ASCs and subsequently hyper-activation of Akt-mTOR drives adipogenesis and premature senescence. Moreover, differentiating ASCs and/or mature adipocytes may acquire features of cellular senescence.

## INTRODUCTION

Obesity is a major public-health problem worldwide. Underlying cause of this epidemic is the inherent expansion and renewal capacity of adipocytes, which differentiate from adipose stromal/progenitor cells (ASCs) in a process referred to as adipogenesis [1-3]. ASCs derived from the stromal-vascular fraction (SVF) of white adipose tissue (WAT) are multipotent and can differentiate *in vitro* into several lineages [2, 4, 5]. Adipogenesis involves ASC determination and terminal differentiation into adipocytes, which express fat cell specific genes and accumulate lipids [6, 7]. This process is governed by a complex signal transducing network including Delta-like protein 1/Preadipocyte factor 1-, Wnt-, insulin-, IGF-1-signaling and numerous additional pathways, which eventually activate or repress a cascade of adipogenic transcription factors. Most central in this cascade are members of the CCAAT/enhancer-binding protein (C/EBP) family and the nuclear receptor peroxisome proliferator-activated receptor-γ2 (PPARγ2). Weight-loss (WL) leads to a reduction in the size of adipocytes. This is associated with endocrine and metabolic benefits [8]. Health-promoting effects of WL exceed however those directly associated with reduction of adipocyte size and fat mass [9]. Increasing evidence suggest beneficial effects of WL on ASCs [10, 11]. We have recently demonstrated that WL leads to upregulation of the small GTPase, GTP-binding RAS-like 3 (DIRAS3) [12, 13], in ASCs of human subcutaneous (s) WAT [14]. DIRAS3 negatively regulates adipogenesis via inhibition of Akt–mechanistic target of rapamycin (mTOR) signaling in the ASCs [14]. Akt-mTOR signaling is well-known as positive regulator of adipogenesis and inhibition of this pathway protects from obesity [15]. This underscores the role of DIRAS3 as WL target gene.

Obesity is associated with an increased number of senescent ASCs [16, 17]. Reduced Akt-mTOR signaling decreases cellular senescence [18] and induces lifespan extension in animal models [15]. We have previously shown that long-term WL postpones replicative senescence in human ASCs [10]. Whether the WL target gene DIRAS3 is involved in regulation of proliferation and senescence in ASCs is unknown. Cellular senescence plays an important role in tumor suppression and organismal aging and emerging evidence suggest additional relevance in development, tissue remodeling and repair [19]. Cellular senescence results in terminal cell cycle arrest eventually induced by up-regulation of the cell cycle inhibitors p16^INK4A^ and p21^CIP1^ and represents phenotypically diverse cellular states, which are characterized by distinct morphological and biochemical alterations [19]. The senescence program can be induced by various cell-intrinsic and -extrinsic stress stimuli, for example DNA damage, oncogene assault induced by activation of oncogenes [20] or loss of tumor suppressors [21] and inflammation [22]. In aged tissues, including sWAT, senescent cells accumulate, which exacerbate dysfunction and contribute to the aging phenotype [16, 23]. Ablation of senescent cells in aged sWAT of mice alleviates age-related dysfunctions [24-26]. These findings underscore the importance of cellular senescence in adipose tissue aging and are reflected by progenitor cell populations isolated from adipose depots of older donors, which exhibit impaired replicative and adipogenic capacity and contain senescent cells [25, 27-29]. The mechanisms underlying senescence in ASCs are however not precisely understood. In the present study, we investigated the influence of DIRAS3 on cellular senescence and proliferative capacity of ASCs of the human sWAT.

## RESULTS

### DIRAS3 suppresses hyper-activation of Akt-mTOR pathway and sustains proliferation of human ASCs

**Figure 1:**
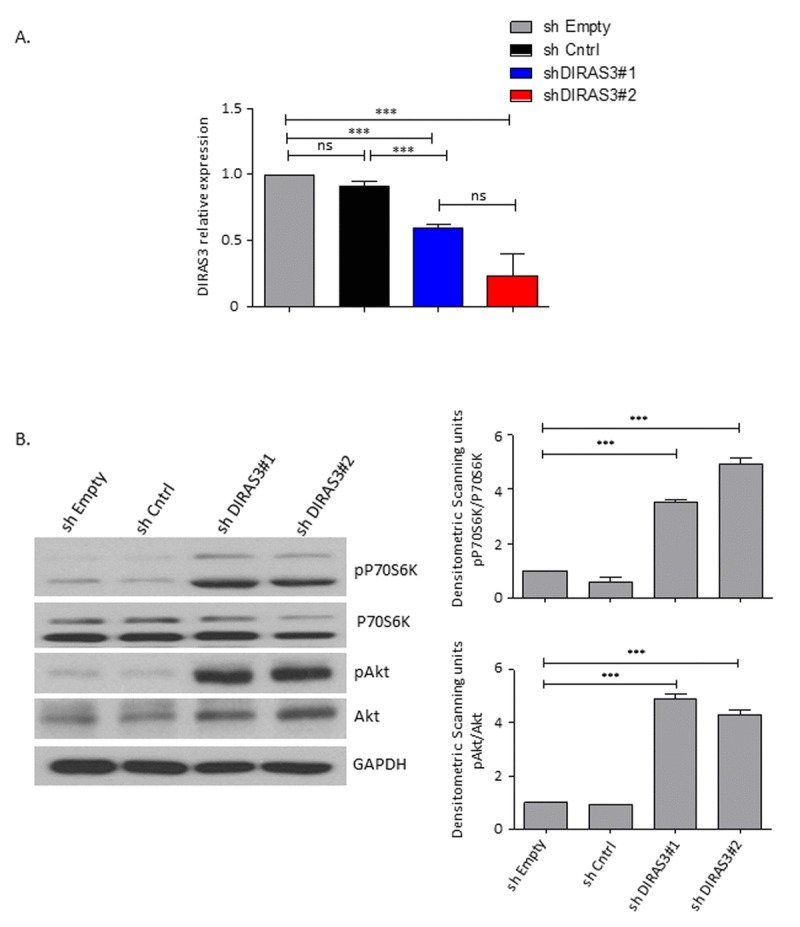
DIRAS3 knock-down (KD) leads to an upregulation of Akt-mTOR signalling in proliferating ASCs (**A**) Efficiency of DIRAS3 KD in ASCs. DIRAS3 mRNA levels were measured by quantitative real time PCR (q-RT PCR) following infection of ASCs with lentiviruses expressing DIRAS3 specific shRNA. An empty vector control (shEmpty) and a scrambled sh sequence (shCntrl) are employed as controls (n=4). **(B)** Activation of Akt and p70S6K by DIRAS3 KD in ASCs. (Left panel) DIRAS3 was KD in ASCs using specific shRNAs as indicated. Cells were starved by serum withdrawal for 48 hours. Afterwards cell lysates were prepared and phosphorylation of Akt (S473) and P70S6K (T389) was examined by immune-blotting using specific antibodies. GAPDH served as an input control. (Right panels) Fold changes in densitometric band intensities presented as Arbitrary Units (AU) for phosphorylated proteins normalized to un-phosphorylated total proteins, acquired by image J were compared. Band intensity of shCntrl 0% FCS was taken as 1. Western blot shown is from replicate from one donor with similar protein expression pattern was observed with 2 different donors. All error bars represents the means ± SEM. p values * = p<0.05, **= p<0.001 and *** = p<.0001.

**Figure 2:**
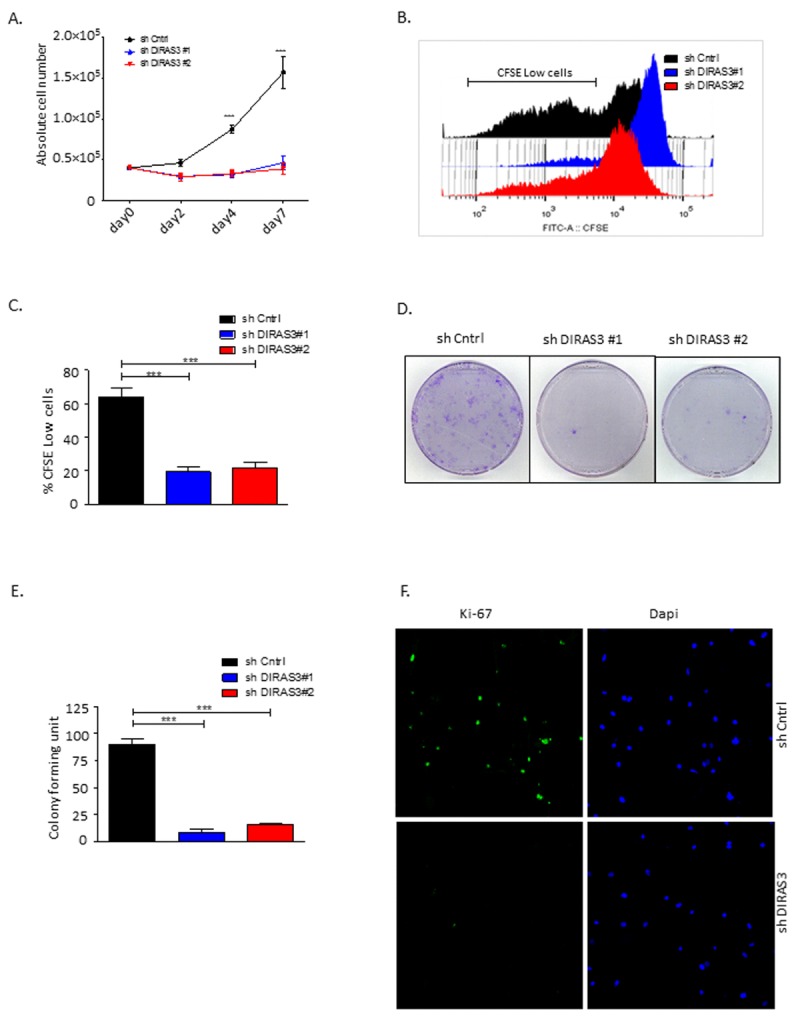
Silencing of DIRAS3 reduces proliferation and self-renewal of human ASCs (**A**) Proliferation of ASCs following knock-down (KD) of DIRAS3 was monitored by cell counting (n=4). **(B** and **C)** Monitoring of cell proliferation by CFSE signal dilution technique. Flow cytometric analyses demonstrated a higher proliferation rate of shCntrl relative to shDIRAS3 infected ASCs, reflected by a significantly higher percentage of CFSE low cells compared to shDIRAS3 infected ASCs. Differentially transduced ASCs were stained with CFSE and cultured for 4 days. Dilution of CFSE signal was monitored by FACS (B). Percentage of CFSE low cells was plotted (C) (n=4). **(D** and **E)** ASCs were seeded at a density of 500 cells in tissue culture dish following transduction with shDIRAS3 and shCntrl expressing lentiviruses and selection. Number of colonies were counted 10 days post seeding after staining with crystal violet (D) and plotted (E) (n=3) **(F)** Expression of Ki-67 was assessed in transduced ASCs via confocal microscopy. Cells were fixed on cover slips and stained for Ki-67 (green) and DAPI (blue) for nuclear staining (n=3). All error bars represents the means ± SEM. p values *** = p<.0001.

To investigate the effect of DIRAS3 knock-down (KD) on Akt-mTOR signaling in ASCs we employed lentivirus mediated DIRAS3 specific shRNA (Fig.1A and [14]). DIRAS3 KD leads to increased activity of Akt-mTOR signaling in ASCs (Fig. 1B). As mTOR activity is essential for cell proliferation but a persistent mTOR complex 1 activation leads to exhaustion of stem cells [18, 30], we investigated the effect of DIRAS3 KD on proliferation of ASCs. We found that DIRAS3 KD abrogates ASC proliferation (Fig. 2A – 2C). This effect was dose-dependent (Supplementary Fig. S1A). We observed a significantly lower colony formation index upon DIRAS3 KD in colony-formation assays (Fig. 2D and 2E). Upon DIRAS3 KD, we detected a strong decrease in the expression of proliferation marker Ki-67, which is expressed in all phases of the cell cycle except G0 (Fig. 2F). We detected neither an increased number of floating cells nor significant difference in percentage of apoptotic cells upon DIRAS3 KD in annexin V/propidium iodide staining assays (Supplementary Fig. S1B), indicating that the lower number of cells and colonies observed upon DIRAS3 KD is not due to increased apoptosis. Together these results indicate that a certain level of DIRAS3 is necessary to suppress hyper-activation of Akt-mTOR pathway and to sustain proliferation, suggesting that DIRAS3 contributes in maintaining self-renewal capacity of the human ASCs.

### DIRAS3 KD induces premature senescence in human ASCs

**Figure 3:**
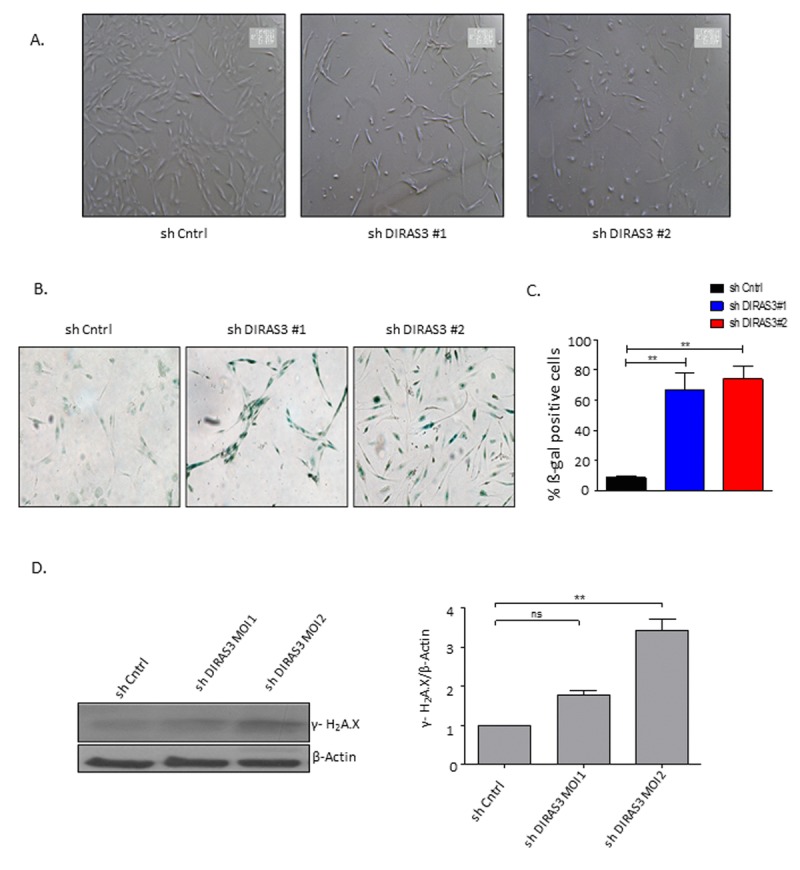

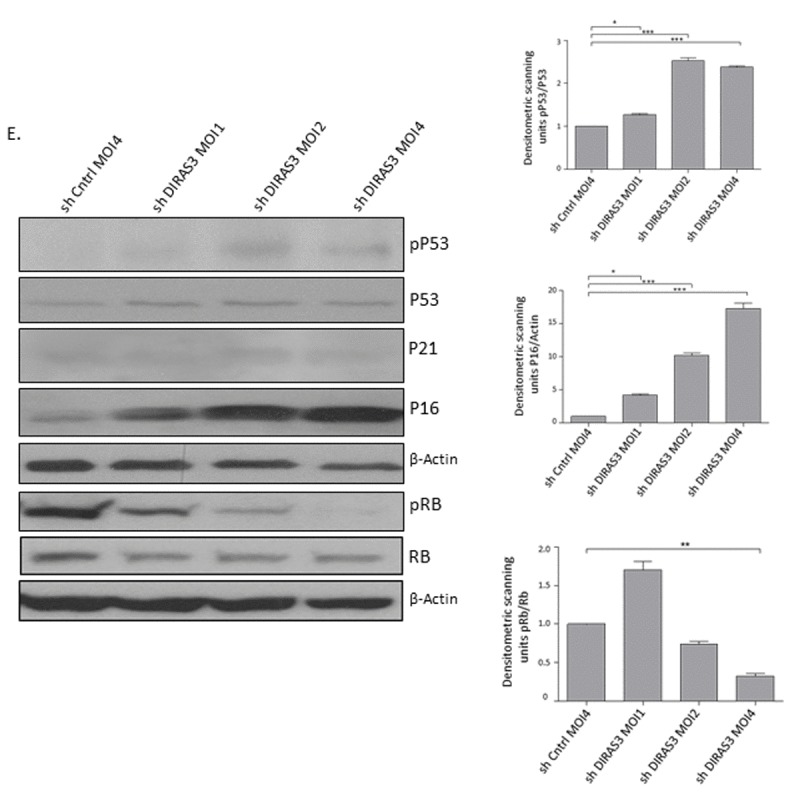
Silencing of DIRAS3 induces premature senescence in human ASCs (**A**) Morphology of ASCs infected with shCntrl and shDIRAS3 was documented using light microscope at 40x magnification. **(B** and **C)** ASCs infected with either shDIRAS3 or shControl (shCntrl) expressing lentiviruses were fixed and stained for SA-β-GAL. Percentage of SA-β-GAL positive cells was calculated by scanning 5 low-power magnification fields (n=3). **(D)** ASCs were transduced by indicated lentiviruses with increasing MOI and cell lysates immunoblotted using phospho-Ser-139 Gamma H2A.X antibody. β-Actin served as a loading control. DIRAS3 was KD in ASCs using lentiviruses expressing specific shRNA at increasing MOI. Cell lysates were immunoblotted with the specific antibodies to investigate accumulation of senescent associated proteins p16^INK4A^, p21^CIP1^, p53 phosphorylated p53 (S15), Rb and pRb (S807/811). β-Actin served as a loading control. Fold changes in densitometric band intensities for phosphorylated proteins normalized to un-phosphorylated total proteins, acquired by image J were compared. Band intensity of shCntrl was taken as 1. Western blot shown is from replicate from one donor with similar protein expression pattern was observed with 2 different donors. All error bars represents the means ± SEM. p values * = p<0.05, **= p<0.001 and *** = p<.0001.

DIRAS3 KD cells displayed a cytoplasm with thin and long processes and a significant number of these cells became flat (Fig. 3A and 3B), resembling the morphological features of the premature senescence phenotype induced by oncogenic assault in primary human fibroblasts [20]. As demonstrated by cytochemical staining (Fig. 3B and 3C) and FACS analyses using a fluorogenic substrate C_12_FDG (Supplementary Fig. S2A), DIRAS3 KD led to a significant increase in the number of senescence-associated *β*-galactosidase (SA-*β*-GAL) positive ASCs and to a significant increase in cell size (Supplementary Fig. S2B). To assess the involvement of DNA damage response (DDR) in DIRAS3 KD induced premature senescence, we analysed formation of senescence-associated heterochromatic foci (SAHF) using immunofluorescence staining and observed higher specific foci in DIRAS3 KD cells (Supplementary Fig. S2C). In addition, we detected a significant higher level of phosphorylated (ser139) γ-H2A.X protein by western blotting in cell lysates from DIRAS3 KD ASCs compared to shCntrl transduced ASCs (Fig. 3D). Cellular senescence can mainly be induced by activation of either p53/p21CIP1, p16INK4A/ Retinoblastoma protein (Rb) or both pathways depending on the nature and magnitude of stress [19]. We detected significant higher levels of phosphorylated (ser15) p53 upon DIRAS3 KD in the ASCs (Fig. 3E), indicating activation of the p53 pathway. Although we found slightly higher p21CIP1 mRNA expression in DIRAS3 KD ASCs relative to shCntrl cells (Supplementary Fig. S2D), we have not observed a significant change in the p21CIP1 protein level (Fig. 3E). This suggests a relatively weak activation of the p53/p21CIP1 arm in DIRAS3 KD induced cellular senescence (DICS) in cultured human ASCs. While, we detected a very strong induction of p16INK4A at both mRNA and protein level in DIRAS3 KD ASCs (Fig. 3E and Supplementary Fig. S2E). Both cyclin-dependent kinase inhibitors (CDKI's), p21CIP1 and p16INK4A, induce proliferation arrest via the inhibition of G1/S CDKs. This leads to activation of cell cycle break Rb by reduced phosphorylation. We analyzed whether DIRAS3 KD leads to reduced Rb phosphorylation. We observed a significant decrease in serine 807/811 phosphorylated Rb in DIRAS3 KD ASCs (Fig. 3E), suggesting that DIRAS3 KD induces proliferation arrest by abrogating Rb phosphorylation.

### DIRAS3 KD ASCs develop a senescence-associated secretory phenotype

**Figure 4:**
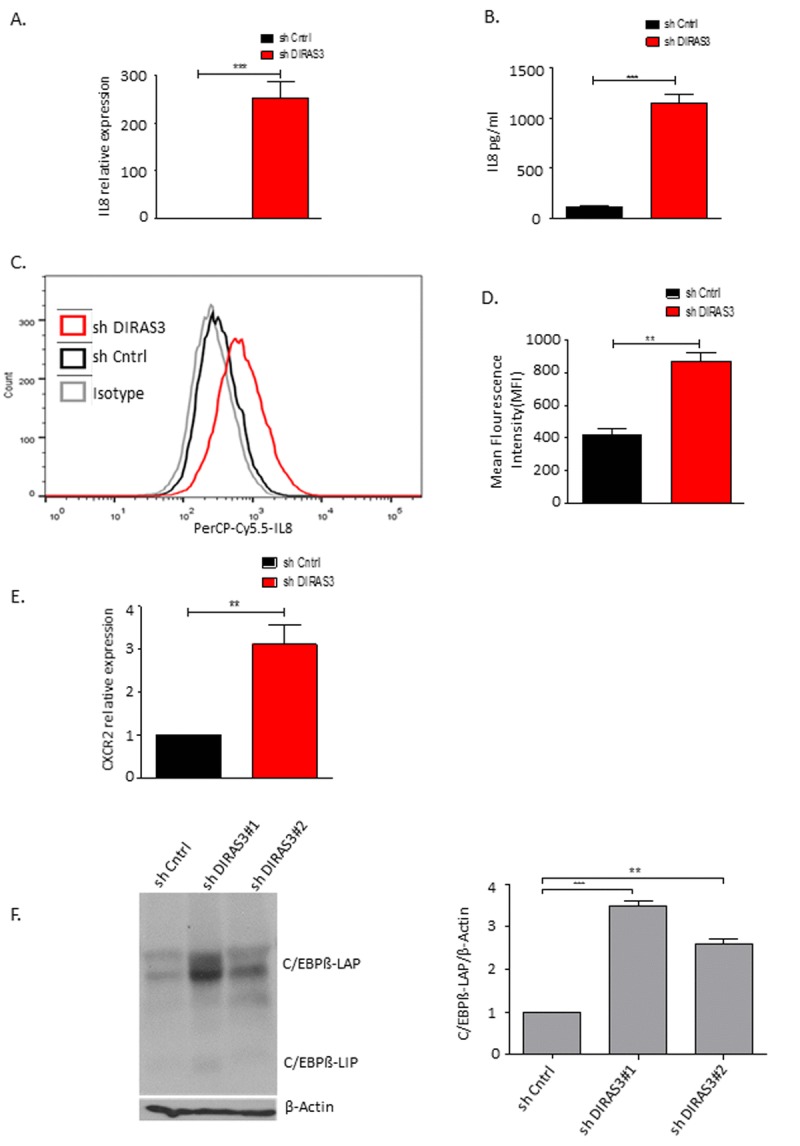
DIRAS3 KD induced senescence lead to higher production of IL-8 (**A**) IL-8 mRNA was quantified using q-RT PCR upon DIRAS3 KD (n=4). **(B)** IL-8 protein level in cell culture supernatant was quantified by ELISA (n=3). **(C** and **D)** Differentially transduced ASCs were incubated for 4 hours with brefeldin A (5μg/ml) – a protein transport inhibitor from Endoplasmic reticulum to Golgi complex, followed by fixation and permeabilization. Cells were stained by PerCP-Cy5.5-IL-8 antibody and analyzed by FACS (n=2). **(E)** CXCR2 mRNA was quantified using q-RT PCR upon DIRAS3 KD (n=4) **(F)** (Left panel) C/EBPβ protein level upon KD of DIRAS3 was analyzed by immune-blotting using anti-C/EBPβ antibodies. β-Actin served as input control. (Right panel) Fold changes in densitometric band intensities for phosphorylated proteins normalized to un-phosphorylated total proteins, acquired by image J were compared. Band intensity of shCntrl was taken as 1. Western blot shown is from replicate from one donor with similar protein expression pattern was observed with 2 different donors. All error bars represents the means ± SEM. p values * = p<0.05, **= p<0.001 and *** = p<.0001.

To analyse whether DIRAS3 KD ASCs show features of the senescence-associated secretory phenotype (SASP) we measured the expression of f interleukin 6 (IL-6), IL-8, IL-1β and TNF α genes coding for secreted factors which are frequently upregulated in senescent cells [31]. Results revealed a strong up-regulation of IL-8 mRNA expression (Fig. 4A) while the expression of the other genes was not increased. PCR data were verified by quantitative ELISA (Fig. 4B) and FACS analysis (Fig. 4C and 4D), showing increased IL-8 staining in DIRAS3 KD ASCs and increased secretion of the pro-inflammatory cytokine from these cells. Acosta et al., 2008 [22] have shown that senescent cells activate a self-amplifying secretory program in which ligands of the IL-8 receptor (CXCR2) reinforce growth arrest. To strengthen the results we analysed CXCR2 expression. We observed a significant increase in the expression of CXCR2, in DIRAS3 KD cells (Fig. 4E).

Mechanistically an up-regulation of C/EBPβ was shown to play a critical role in OIS related induction of IL-8 [22]. Similarly, we found that C/EBPβ protein level is up-regulated in DIRAS3 KD ASCs (Fig. 4F).

### Human DIRAS3 KD ASCs develop a senescence phenotype in posterior sWAT of SCID mice

**Figure 5:**
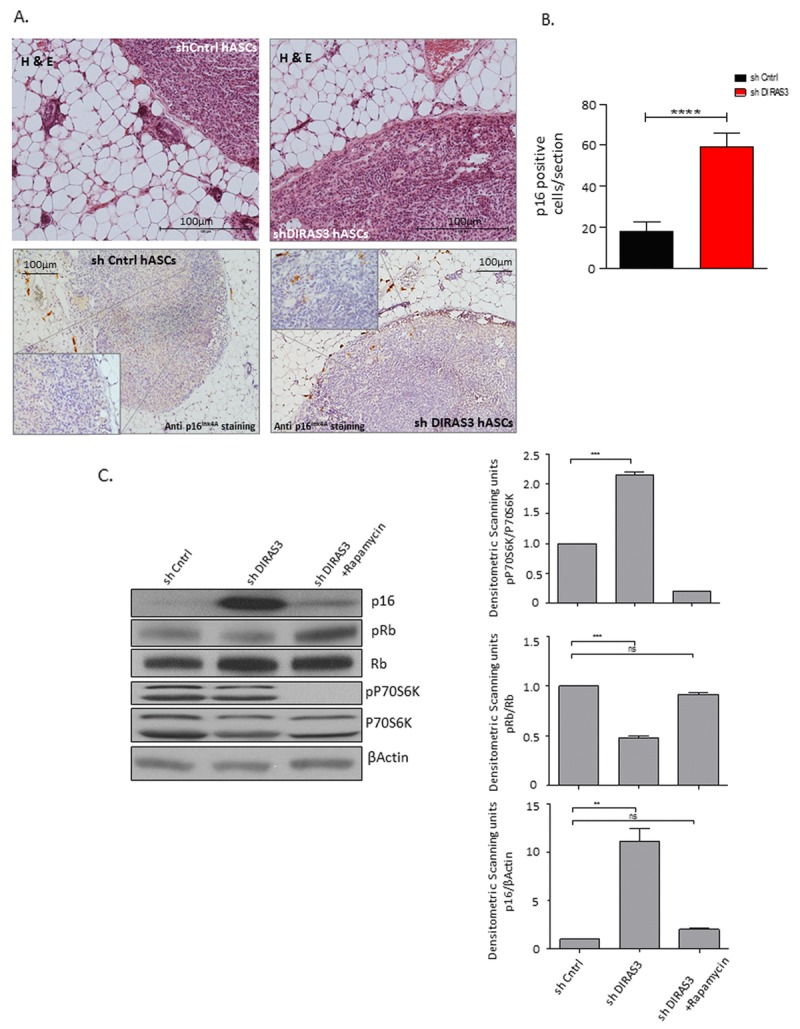
Human DIRAS3 KD ASCs develop a senescence phenotype in posterior sWAT of SCID mice (**A**) (Upper panel) Human shDIRAS3 ASCs (shDIRAS3 hASCs) and shCntrl hASCs were xenotransplanted into posterior sWAT of SCID mice. Injection sites of hASCs were histologically identified and marked by H&E staining. (Lower panel) Senescent DIRAS3 KD hASCs were detected by immunohistochemical staining using anti p16^INK4A^ antibodies. Region of Interest (ROI) is shown in higher magnification. **(B)** Number of p16^INK4A^ positive hASCs per section were counted and plotted (n = 5 per group). **(C)** (Left panel) Cell lysates from control ASCs and DIRAS3 KD ASCs cultured with and without 20 nM rapamycin were blotted for p16^INK4A^, Rb, pRb (S807/811), P70S6K and pP70S6K (T389) using specific antibodies. β-Actin served as loading control. Note, rapamycin was added 2 days after virus infection. (Right panels) Fold changes in densitometric band intensities presented as Arbitrary Units (AU) for phosphorylated proteins normalized to un-phosphorylated total proteins, acquired by image J were compared. Band intensity of shCntrl was taken as 1 (n=2). All error bars represents the means ± SEM. p values * = p<0.05, **= p<0.001 and *** = p<.0001.

Since a DIRAS3 ortholog does not exists in mice [12], we analysed the *in vivo* relevance of DICS in a SCID mouse xenograft model. DIRAS3 KD and shCntrl human ASCs were injected into the posterior sWAT of SCID mice and tissue sections harboring the injection site were stained by immunohistochemistry for p16^INK4A^ at six weeks post-injection. Analyses revealed a significant higher number of p16^INK4A^ positive cells in mice injected with shDIRAS3 transduced ASCs (Fig. 5A and 5B), suggesting that DIRAS3 KD induces senescence in human ASCs in sWAT of mammals. To finally validate our hypothesis that DIRAS3 KD induced hyperactivity of mTOR leads to p16^INK4A^ induction and hence premature senescence, we down-modulated mTOR activity in DIRAS3 KD ASCs using rapamycin. Western blot analyses revealed that rapamycin treatment rescued DIRAS3 KD induced p16^INK4A^ up-regulation and Rb phosphorylation thus supporting our hypothesis (Fig. 5C).

### Silencing of DIRAS3 in ASCs induces both adipogenic differentiation and premature senescence

**Figure 6:**
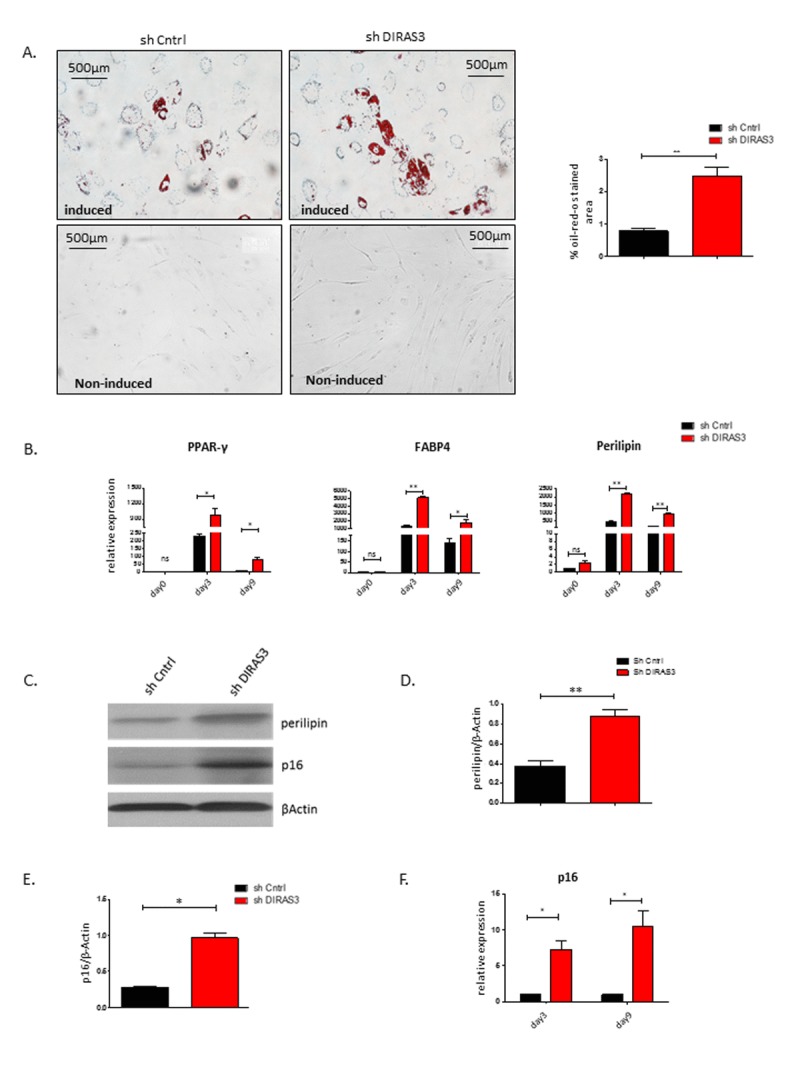
Silencing of DIRAS3 in ASCs induces both premature senescence and adipogenic differentiation (**A**) (Left panel) Adipogenic differentiation of ASCs was estimated by staining the cells with Oil-Red-O stain at day 9 post induction of differentiation. Non-induced ASC controls are shown. (Right panel) Quantification of Oil-Red-O stained area using image J is shown (n=3). **(B)** Expression of PPAR*γ*2, FABP4 and Perilipin mRNA was estimated at day 3 and day 9 post induction of adipogenesis. Expression at day 0 before induction was taken as 1 and fold increase was calculated. Values are normalized to β Actin. **(C)** Perilipin and p16^INK4A^ protein levels were analysed in DIRAS3 KD and control ASCs at day 9 post induction of adipogenesis by western blotting. β-Actin protein served as input control. **(D** and **E)** Densitometric evaluation of western blots bands from figure C. Fold changes in densitometric band intensities of perilipin (D) and p16^INK4A^ (E) normalized to β-Actin protein, acquired by image J were plotted. **(F)** p16^INK4A^ mRNA expression was analysed in DIRAS3 KD (red) and control ASCs (black) at day 3 and 9 post adipogenesis induction by q-RT_PCR analysis. All error bars represents the means ± SEM. p values * = p<0.05, **= p<0.001 and *** = p<.0001.

We have recently demonstrated that silencing of DIRAS3 induces adipogenic differentiation in ASCs via activation of Akt-mTOR signaling [14]. DIRAS3 KD strongly activated this signaling pathway and facilitated adipogenesis in ASCs treated with differentiation hormone cocktail. In the present study, we show that DIRAS3 KD mediated activation of Akt-mTOR signaling induces proliferation arrest and premature senescence in normal proliferating ASCs. To better understand the relationship between the role of DIRAS3 in regulation of senescence and adipogenesis we induced adipogenic differentiation in DIRAS3 KD ASCs and analysed senescence, adipogenesis and adipocyte markers 3 and 9 days after induction of differentiation. As shown before, silencing of DIRAS3 enhanced the induction of adipocyte differentiation as demonstrated by a higher number of cells staining positive by Oil-Red-O staining (Fig. 6A). Furthermore, we observed a significant up-regulation of the expression of the key adipogenic transcription factor PPARγ2 and the adipocyte markers FABP4 and perilipin (Fig. 6B). Moreover, the level of the major adipocyte marker protein perilipin was increased in the resulting adipocytes (Fig. 6C and 6D). Intriguingly, DIRAS3 KD resulted also in strong upregulation of the major senescence marker p16^INK4A^ at mRNA (Fig. 6F) and protein level (Fig. 6C and 6E) in the resulting DIRAS3 KD adipocytes. Furthermore, p16^INK4A^ protein was also detectable in *in vitro* differentiated adipocytes arising from wild type ASCs (Fig. 6C). Double staining experiments of *in vitro* differentiated adipocytes arising from ASCs 14 days after induction of adipogenic differentiation showed that these cells are SA-β-galactosidase positive and contain Oil-Red-O stained fat droplets (Supplementary Fig. S3). In conclusion, these data indicate that silencing of DIRAS3 in human ASCs stimulates both adipogenic differentiation and premature senescence. Moreover, our data suggest that wild type adipocytes per se show features of cellular senescence.

## DISCUSSION

Deterioration of sWAT is a common feature with advancing age [27, 32]. The age-related detrimental changes in adipose tissue are not well understood. The current model suggests that accumulation of senescent adipose progenitors in aged WAT, which exacerbate dysfunctions and loss of fat mass, contributes to this aging phenotype [16, 23-26]. Adipose tissue is also a site of senescent cell accumulation in obesity [16,17]. In fact, while the ASC pool capable of self-renewal and differentiation is essential for homeostasis, regeneration, expansion and, hence functional maintenance of WAT, ASC populations isolated from adipose depots of aged or obese donors exhibit impaired replicative and adipogenic capacity and contain senescent cells [10, 16, 17, 25, 29].

Little is known regarding the molecular mechanisms underlying cellular senescence in ASCs. We recently identified the small GTPase, DIRAS3, a well characterized tumor suppressor protein [13], as a WL target gene in ASCs of human sWAT and show that DIRAS3 negatively regulates adipogenesis [14]. In the present study, we showed for the first time that silencing of DIRAS3 induces cell cycle arrest and premature senescence in ASCs by hyperactivation of mTOR. Our results indicate that this leads eventually to premature senescence by strong activation of the p16^INK4A^/Rb pathway and to a lesser extent of the p53/p21^CIP1^ pathway. DDR likely contributes to this senescence phenotype, as DIRAS3 KD ASCs showed an increased number of foci containing the phosphorylated form of histone H2AX, a marker of DNA damage.

Studies support the hypothesis that signaling pathways that induce stem/progenitor cell growth, and hence transient amplification, contribute to stem cell exhaustion and depletion when aberrantly or persistently activated (see [33] for a review). Thus, an appropriate intensity of growth-regulatory pathways, such as mTOR signaling, is important for decisions on given cell fates, such as self-renewal, proliferation, senescence and differentiation in adult stem/progenitor cells. Our findings are in keeping with this model. DIRAS3 silencing induced hyperactivity of mTOR promotes adipogenesis and premature senescence in human ASCs (this study, [14]). Rapamycin abrogates these effects. Similar data are available from other adult stem cell models. Epithelial stem cells undergo differentiation and senescence upon aberrant stimulation of mTOR [18], while reducing mTOR activity by rapamycin prevents senescence and terminal differentiation [30]. Aging adult muscle stem cells (MuSCs) can shift from a quiescent phenotype, which is crucial for transient amplification and self-renewal, to an over-activated phenotype characterized by increased activity of different signaling cascades that leads to premature senescence and terminal differentiation [34-36]. Moreover, mTOR acts as a regulator of the balance between quiescence, senescence and differentiation in neural stem cells [37, 38]. Precedence from work on MuSCs suggests that intrinsic mediators of stem cell quiescence exists, which can counteract the senescence program and promote self-renewal [39]. DIRAS3 as an intrinsic negative regulator of Akt-mTOR signaling may fulfill a similar function in ASCs, namely negative regulation of terminal adipogenic differentiation and senescence driven by stimulation of the Akt-mTOR pathway.

Senescent cells develop a SASP including chemo/cytokines with pro-inflammatory properties, which modulate tissue microenvironment and systemic phenotypes [31, 40]. The present study showed that DICS contributes to the manifestation of a SASP in ASCs and identified IL-8 as a specific factor up-regulated by DICS. It is interesting to note that IL-8 is specifically upregulated in senescent ASCs isolated from omental adipose biopsies of severe obese humans [17], because we previously identified DIRAS3 as a WL target gene in ASCs of formerly obese people [14]. We found no upregulation of IL-6, IL-1β and TNF α by silencing of DIRAS3. Others have shown that for example IL-6 is increased in p16-positive cells isolated from inguinal adipose tissue of BubR1 progeroid mice [24]. Thus the composition of the SASP varies dependent on the senescent cell type, tissue and stress-inducing stimuli.

As mentioned above, ample evidence suggests that impaired replicative and adipogenic capacity are hallmarks of preadipocyte aging and adipogenesis is impaired in senescent ASCs. Senescent cells leave the cell cycle with a permanent arrest in G0 and lose their ability to differentiate [19]. Our present study shows that silencing of DIRAS3 in human ASCs activates Akt-mTOR signaling and induces premature senescence. DIRAS3 KD induces however also adipogenesis and the resulting adipocyte population contains increased level of adipocyte and senescence markers. Moreover, we demonstrate that *in vitro* differentiated wild type adipocytes possess characteristics of adipocytes and senescence at the single cell level. This suggests that differentiating DIRAS3 KD ASCs and/or terminally differentiated adipocytes most likely acquire a senescence-like phenotype. Similar to our study it was shown that mature adipocytes of mice on a high-fat diet obtain key characteristics of senescence [16]. Moreover, other post-mitotic, terminally differentiated cells, which cannot re-enter the cell cycle, have also been found to develop characteristics of cellular senescence. This was shown for cardiac myocytes and neurons and is discussed as “senescent after differentiation”, a novel pathway to the formation of senescent cells [41].

The interaction between terminal differentiation and cellular senescence is poorly understood. Silencing of DIRAS3 in ASCs activates Rb induced cell cycle arrest. Rb plays a role in cellular senescence but also in several terminal differentiation programs of adult stem/progenitor cells [42]. Available data on adipogenesis are complicated. Studies in preadipocyte cell lines showed either activation [43] or repression of adipogenesis by Rb [44]. Moreover, Rb was shown to determine white versus beige adipocyte differentiation [45]. More work is necessary to better understand the signaling circuits down-stream of DIRAS3, which regulate adipogenesis and senescence.

Previous work from our group indicated that WL postpones the onset of replicative senescence in ASCs explanted from sWAT of formerly obese humans [10]. Subsequently we identified DIRAS3 as a WL target gene up-regulated in ASCs of sWAT of formerly obese people [14]. In combination with these studies, our present findings suggest that WL-induced upregulation of DIRAS3 reduces senescence and adipogenesis in ASCs of formerly obese people and promotes ASC self-renewal by restricting Akt-mTOR signaling. This is in keeping with the hypothesis that obesity-induced hyperactive Akt-mTOR signaling contributes to premature aging and reduced self-renewal capacity of ASCs while WL inhibits this pathway and hence protects ASCs.

In conclusion, the present study demonstrates a novel function of DIRAS3 in regulation of proliferation, senescence and differentiation of human ASCs.

## METHODS

### Donors

Human sWAT samples were taken from eight formerly obese Caucasian women undergoing routine abdominoplasty after long-term weight loss interventions at the Institute for Plastic and Reconstructive Surgery (Medical University Innsbruck, Austria) [14, 46]. The patients gave their informed written consent and had been approved by the ethical committee of Innsbruck Medical University, Austria, according to the Declaration of Helsinki. None of the women suffered from diabetes, liver, renal or other severe metabolic diseases. None of the women had cancer. The clinical and anthropometric parameters are indicated in Table 1. The sWAT samples were taken from the lower abdomen (infraumbilical). The probes were exactly resected from the layer between fascia of scarpa and rectus fascia.

**Table 1:** Characteristics of the subjects participated in this study

n	Age (Years)	Weight	BMI
8	**35 + 12**	**65 + 6**	**25 + 4**

All donors were healthy formerly obese Caucasian women, which underwent routine abdominoplasty after long-term weight-loss interventions. None of the women suffered from diabetes, liver, renal or other severe metabolic diseases. None of the women had cancer. Clinical and anthropometric parameters are indicated.

### Mouse Xenograft studies

Mice were treated in accordance with the guidelines of the “European Convention for the Protection of Vertebrate Animals used for Experimental and other Scientific Purposes” and the Austrian law. Animal experiments were approved by the ethics committee of the Austrian Federal Ministry of Science and Research (Application No. Zl. 188809/13). Six week old Crl:SHO-*Prdkc^scid^Hr^hr^* female mice were purchased from Charles River (Germany). Human ASCs were infected with shCntrl or shDIRAS3 expressing lentiviruses. Cells were puromycin selected and 4 days later 1.5 × 10^5^ cells were injected subdermally into the flank of each mice (n=5 per group). Six weeks later, Forane® anesthetized mice were sacrificed and transplanted tissue site was surgically removed, fixed, paraffin embedded, sectioned and stained. Tissue sections were selected based on the hematoxylin and eosin stain. Antigen retrieval was performed by boiling in 6.5mM sodium citrate buffer (pH 6) for 10 min. 3%BSA/PBS was used for blocking. Sections were incubated at 4 °C overnight with anti p16^INK4A^ antibody (1:50) (p16 (clone JC8), Santa Cruz). Anti-mouse secondary Ab was applied for 1hr at room temperature. DAB chromogen was added for 1 min, washed followed by hematoxylin staining for 1 min and mounting. Number of p16 positive cells was counted per section.

### Isolation of ASCs from human subcutaneous adipose tissue and cell culture of ASCs

ASCs were isolated from abdominal sWAT samples obtained by incision from female donors undergoing elective plastic abdominal surgery essentially as described [5]. Briefly, after surgery adipose tissue biopsies were transferred into sterile serum-free ASC medium (DMEM/F-12 medium (1:1) with HEPES and L-glutamine (Gibco, Vienna, Austria), supplemented with 33 μM biotin, 17 μM pantothenate and 12.5 μg/ml gentamicin and processed in a laminar flow sterile work bench class II. After rinsing with sterile PBS fibrous material and blood vessels were dissected. The tissue was cut into pieces and digested by collagenase in digestion buffer (PBS containing 200 U/ml collagenase (CLS Type I, Worthington Biochemical Corp., Lakewood, NJ) and 2% w/v BSA). The cells were purified by centrifugation, filtration steps and erythrocyte lysis (erythrocyte lysis buffer was 0.155 M NH4CI, 5.7 mM K2HPO4, 0.1 mM EDTA, pH 7.3). The isolated cells were referred to as stromal vascular fraction (SVF). Afterwards the cells of the SVF were suspended in ASC medium (DMEM/F-12 medium (1:1) with HEPES and L-glutamine (Gibco, Vienna, Austria) containing 33μM biotin, 17μM pantothenate and 12.5μg/ml gentamicin) supplemented with 10% FBS (Gibco, Vienna, Austria) and seeded into 6-well cell culture dishes at a density of 70 000 cells per cm2. Following 16 h of attachment, cells were kept for 6 days in serum-free ASC medium and non-adherent cells were continuously removed by washing. The remaining cell fraction was referred to as passage 1 (P1). These cells were then stored in liquid N2. After thawing cells were seeded overnight in ASC medium supplemented with 10% FBS, adherent cells were washed and cultivated in PM4 medium (ASC medium supplemented with 2.5 % FBS, 10 ng/ml EGF, 1 ng/ml bFGF, 500 ng/ml insulin). Cells were passaged at a ratio of 1:2 when they reached 70% confluence.

### Adipogenesis assay

ASCs were seeded at a density of 10,000 cells/cm2 and grown till confluence in PM4 medium [47]. After a resting period of 48 hours in ASC medium (Dulbecco's modified Eagle medium/F-12 medium (1:1) with hydroxyethylpiperazineethanesulfonic acid and -glutamine (Gibco), supplemented with 33 μM biotin, 17 μM pantothenate, 12.5 μg/mL gentamicin), adipogenesis was induced using differentiation medium (ASC medium supplemented with 0.2nM insulin (Roche, Vienna, Austria), 0.5mM 3-isobutyl-1-methylxan-thine, 0.25 μM dexamethasone, 2.5% fetal bovine serum, and 10 μg/mL transferrin (Sigma, Vienna, Austria). After day 3 of differentiation, the medium was changed and the cells were cultivated in differentiation medium without 3-isobutyl-1-methylxanthine. For optical visualization of lipid droplets, cells were fixed with 4% paraformaldehyde in phosphate buffered saline (PBS) for 1 hour and stained with 0.3% Oil-Red-O (Sigma) in isopropanol/water (60:40) for 1 hour. Final washing procedure was carried out two times with H_2_0.

### Proliferation assay

0.5×10^5^ differentially transduced cells were seeded in 6-well plates and proliferation of cells was monitored by counting the cells over a period of seven days from seeding.

### Colony formation assay

The colony formation assay was done as described [48]. Briefly, 500 cells either shDIRAS3 or shControl transduced were seeded in six well plates. After 10 days of culture colonies derived from single cells were fixed, stained with crystal violet and counted.

### CSFE proliferation assay

Assay was conducted as described [49]. Briefly, ACSs were infected with either shDIRAS3 or shControl expressing lentiviruses and selected using puromycin. Cells were stained with 1μM CFSE in PBS. Cells were incubated for 10min at 4°C and washed twice with 10%FCS/DMEM-F12 HAM to quench unbound CFSE and its deacetylated form. Followed by four days in culture CFSE signal was analyzed by FACS Canto (BD Biosciences).

### Retroviral gene expression system

The retroviral gene expression system used in the present study is described [14, 5]. DIRAS3 shRNA lentiviruses: sh1 (TRCN0000047771) and sh2 (TRCN0000047772) are from the shRNA set #RHS4533 for NM_004675 from Open Biosystems, Vienna. In short, DIRAS3 shRNA and scrambled shRNA (sh Cntrl) cloned in pLKO.1 – TRC cloning vector [50] were purchased from Open Biosytems, Vienna. pLKO.1 empty vector was a gift from David Root (Addgene plasmid # 10878). These vectors were transformed and propagated in One Shot Stbl3 chemically competent *E. coli* (Invitrogen, USA). For lentivirus particle production each sh plasmid was separately co-transfected with packaging plasmid pMD2.G and psPAX2 (Addgene plasmids 12259 and 12260) into HEK293T cells, as described [5]. Briefly, 9 million HEK293T cells in 10 ml DMEM medium (Sigma) with 10 % FCS (Sigma, Vienna, Austria) were transfected with 4.4 μg sh plasmid, 11 μg psPAX2 and 3.6 μg pMD2.G plasmid using 54 μl Lipofectamine 2000 (Life Technologies). After 16 hours medium was replaced with 6 ml fresh DMEM medium containing 10 % FCS. After 48 hours virus particles were harvested by collecting the supernatant and centrifuging at 3000 rpm for 10 min. The supernatant was syringed filtered through 0.45μm filter, aliquoted and stored at −80°C. Transduction efficiency of lentiviruses in human ASCs was confirmed by transducing these cells with lentiviruses expressing GFP. The efficiency of infection was > 90 % (Supplementary Fig. S1C).

### SA-β-Galactosidase staining of senescent cells

The senescent status of cells was verified by in situ staining for SA-β-galactosidase, as described [29]. Briefly, cells grown on 9.6-cm2 cell culture dishes were washed three times with PBS and fixed with 2% formaldehyde/0.8% glutaraldehyde in PBS. After washing with PBS, the cells were incubated in β-galactosidase staining solution (150mM NaCl, 2mM MgCl2, 5mM potassium ferricyanide, 5mM potassium ferrocyanide, 40mM citric acid, 12mM sodium phosphate, pH 6.0, containing 1mg/mL 5-bromo-4-chloro-3-indolyl-β-d-galactoside (X-gal)) for 24 hours at 37°C. The reaction was stopped by washing in PBS. The fluorescence based SA-β-Galactosidase staining assay was performed as described by [51]. Briefly, cells were incubated with 100nM bafilomycin A1 for 1 h to alkalize lysosomes. Cells were further incubated for 2 h with C_12_FDG at a final concentration of 33μM, trypsinized, washed and analyzed by FACS.

### Laser scanning confocal indirect immunofluorescence microscopy (IF-CLSM)

IF-CLSM was performed as described [5]. Antibodies against Ki - 67 (Neomarkers) and gamma H2A.X (Phospho S139) (Abcam) were used in combination with Alexa Fluor 488 goat anti rabbit and Alexa Fluor 488 donkey anti mouse (Invitrogen) antibodies.

### Quantitative RT PCR gene expression analysis

The method is described [5]. Primers sequences are shown in Table 2.

**Table 2:** The primer sequences used the present study are indicated

Gene	Direction	Sequence
βActin	Forward Reverse	AGAAAATCTGGCACCACACC AGAGGCGTACAGGGATAGCA
DIRAS3	Forward Reverse	CATAAGTTCCCCATCGTGCT GAACAGCTCCTGCACATTCA
p16	Forward Reverse	CCCCCACTACCGTAAATCTCCAT CTGCCATTTGCTAGCAGTGTGACT
P21	Forward Reverse	AGACCAGCATGACAGATTTC ACTGAGACTAAGGCAGAAGA
IL-8	Forward Reverse	AAGGAAAACTGGGTGCAGAG ATTGCATCTGGCAACCCTAC
FABP4	Forward Reverse	CAGTGTGAATGGGGATGTGA CGTGGAAGTGACGCCTTT
PPARγ2	Forward Reverse	ATGGGTGAAACTCTGGGAGA TGGAATGTCTTCGTAATGTGGA
Perilipin	Forward Reverse	GACAACGTGGTGGACACAGT CTGGTGGGTTGTCGATGTC

### Western blot analysis

Western blot analysis was performed as described [5]. Cell lysates were separated by SDS-PAGE and blotted on PVDF membranes. Following antibodies were used: Mouse anti human β-Actin, GAPDH (Ambion), CEBP-beta (C-19) (Santa Cruz), gamma H2A.X (Phospho S139) (Abcam), anti-mouse IgG HRP conjugate (Promega, Mannheim #W420B), donkey anti goat IgG-HRP (Promega, Mannheim #V805A), rabbit anti-rat IgG HRP (Dako Cytomation, Hamburg, #P0450), pP53, P53, pRb, Rb, pAkt (S473), Akt, pP70S6K (T389) and P70S6K, were from (Cell Signaling). Image J software was used for densitometric analyses.

### Flow cytometry analysis

Differentially transduced ASCs were incubated for 4 hrs with brefeldin A (5μg/ml), followed by fixation and permeabilization. Cells were stained by PerCP-Cy5.5-IL-8 antibody and analyzed by FACS using a FACSCanto II (BD Biosciences). Data was analyzed employing FlowJo software.

### IL-8 ELISA

ELISA was performed following the protocol from Human IL-8 (2^nd^ Generation) ELISA Ready-SET-Go kit (eBioscience Ref: 88-8086-22).

### Statistical Analysis

A statistical analysis was performed in GraphPad Prism (GraphPad Software Inc., La Jolla, CA, USA). The significance of difference between means was assessed by Student's t test or analysis of variance (ANOVA). Error bars are represented as the mean ± SEM.

## SUPPLEMENTARY MATERIALS FIGURES


