# Supplementary material for: Silencing of the small GTPase DIRAS3 induces cellular senescence in human white adipose stromal/progenitor cells
Source: Aging (Albany NY). 2017 Mar 17;9(3):860–76. doi: 10.18632/aging.101197 (PMC5391236; doi:10.18632/aging.101197)
Supplement: Supplementary file 1 [file aging-09-860-s001.pdf]

SUPPLEMENTARY MATERIALS

Supplemental Figures.

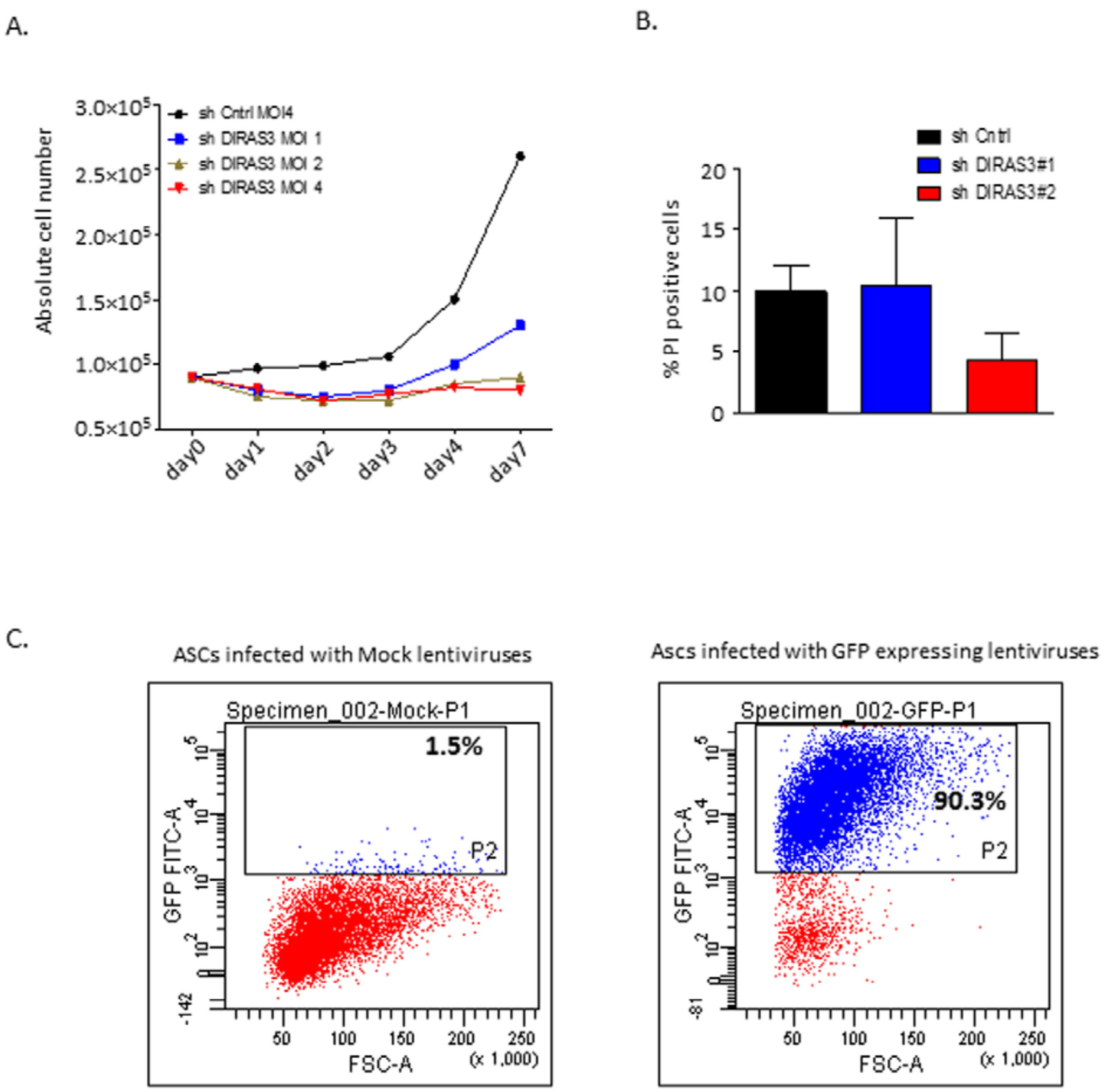

Supplemental Figure S1. Supplementary experiments analysing the role of DIRAS3 in human ASC proliferation and cell death. (**A**) Passage 4 ASCs were infected with increasing Multiplicity of Infection (MOI) of lentiviruses expressing shDIRAS3 #2. Cells were seeded at equal number and counted after harvesting over a period of 7 days. **(B)** Percentage of apoptotic ASCs was estimated employing propidium iodide staining by FACS. All error bars represents the means ± SEM. p values * = p<0.05, **= p<0.001 and *** = p<.0001. **(C)** ASCs were infected with lentiviruses expressing GFP, MOI 2. The transduction efficiency was analysed by analysing the % GFP positive cells by FACS compared to ACSs infected with Mock lentiviruses.

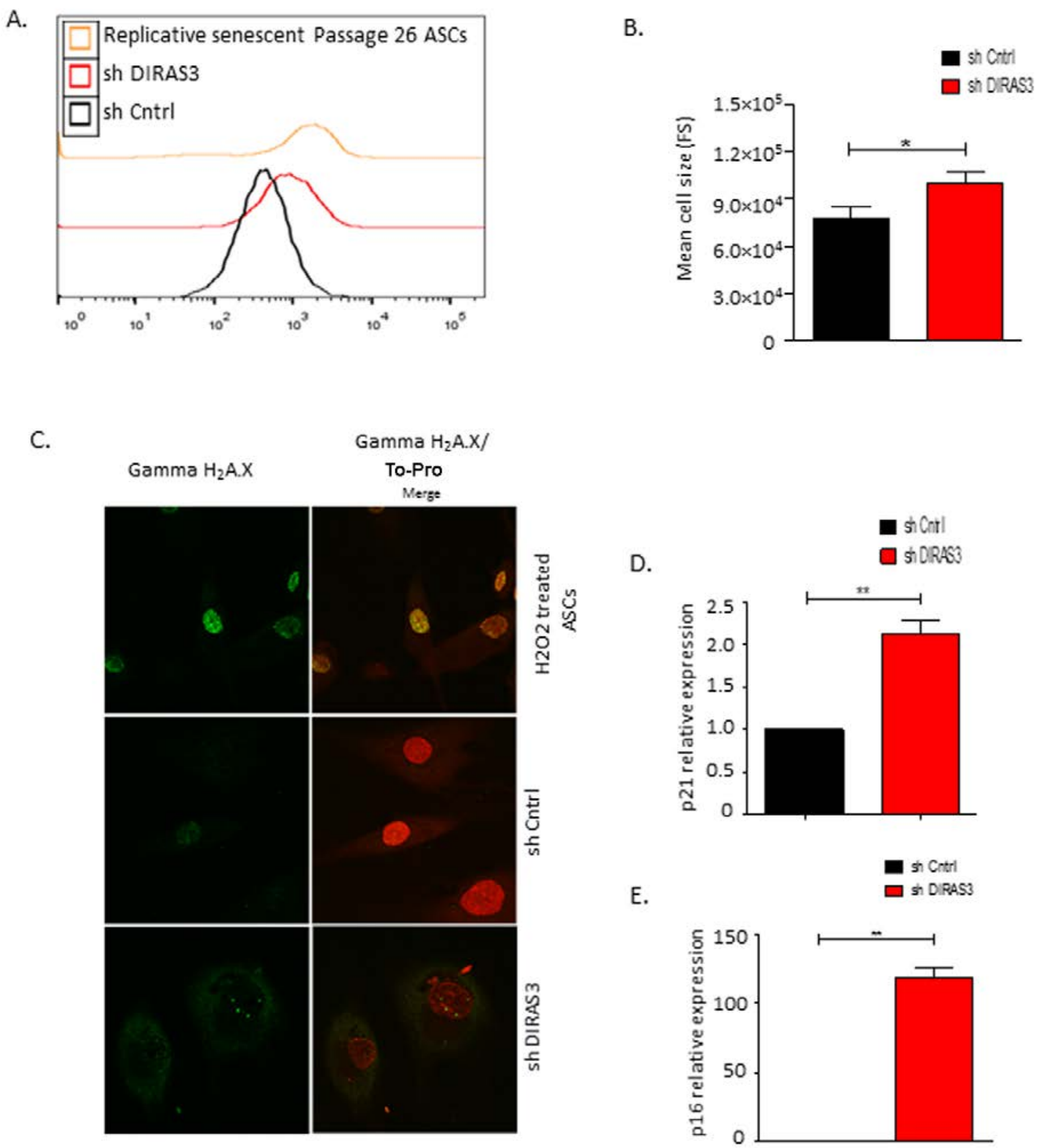

Supplemental Figure S2. Supplementary experiments analysing the role of DIRAS3 in senescence of human ASCs. (**A**) SA-β-GAL positive cells were analysed by FACS using C_{12}FDG as a substrate. Mean fluorescence intensity was measured as a parameter of SA-β-GA activity. **(B)** Mean cell size (forward scatter value as a measure of cell size index) of ASCs was determined by FACS following DIRAS3 knock-down using sh DIRAS3 # 2. **(C)** shDIRAS3 or shCntrl transduced ASCs were immuno-stained with phospho-Ser-139 Gamma H2A.X antibody (green) and To-Pro 3 (red) for nuclear staining and visualized via confocal microscopy. H2O2 treated ASCs were used as a positive control. **(D** and **E)** mRNA expression levels of p21CIP1 (**D**) and p16INK4A (**E**) were analyzed by q-RT PCR. All error bars represents the means ± SEM. p values * = p<0.05, **= p<0.001 and *** = p<.0001.

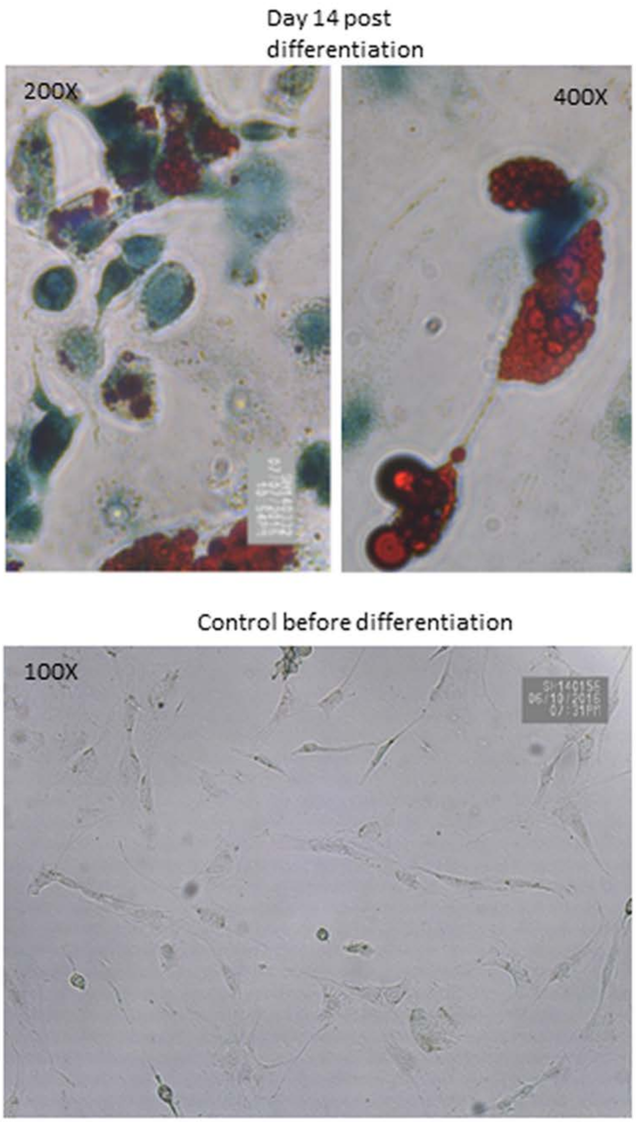

Supplemental Figure S3. *In vitro* differentiated adipocytes are SA-β-galactosidase positive and contain Oil-Red-O stained fat droplets. Adipogenesis was induced in density arrested passage 4 ASCs (lower panel) by adding adipogenic differentiation cocktail. 14 days later cells were double stained by Oil-Red-O (red) to detect fat droplets and for the senescence marker SA-β-galactosidase (blue) (upper panels). Magnifications are indicated.
